# Coordinative Compounds Based on Unsaturated Carboxylate with Versatile Biological Applications

**DOI:** 10.3390/molecules29102321

**Published:** 2024-05-15

**Authors:** Gina Vasile Scaeteanu, Mihaela Badea, Rodica Olar

**Affiliations:** 1 Department of Soil Sciences, University of Agronomic Sciences and Veterinary Medicine, 59 Mărăști Str., 011464 Bucharest, Romania; gina.scaeteanu@agro-bucuresti.ro; 2 Department of Inorganic and Organic Chemistry, Biochemistry and Catalysis, Faculty of Chemistry, University of Bucharest, 90–92 Panduri Str., S5, 050663 Bucharest, Romania; mihaela.badea@chimie.unibuc.ro

**Keywords:** biofilm, complex, N-based heterocycle, planktonic, resistance, unsaturated carboxylate

## Abstract

This review presents an overview of the biological applications of coordinative compounds based on unsaturated carboxylates accompanied by other ligands, usually N-based heterocyclic species. The interest in these compounds arises from the valuable antimicrobial and antitumor activities evidenced by some species, as well as from their ability to generate metal-containing polymers suitable for various medical purposes. Therefore, we describe the recently discovered aspects related to the synthesis, structure, and biological activity of a wide range of unsaturated carboxylate-containing species and metal ions, originating mostly from 3d series. The unsaturated carboxylates encountered in coordinative compounds are acrylate, methacrylate, fumarate, maleate, cinnamate, ferulate, coumarate, and itaconate. Regarding the properties of the investigated compounds, it is worth mentioning the good ability of some to inhibit the development of resistant strains or microbial biofilms on inert surfaces or, even more, exert antitumor activity against resistant cells. The ability of some species to intercalate into DNA strands as well as to scavenge ROS species is also addressed.

## 1. Introduction

Lately, there have been significant research efforts in the fields of both polymers and metal-containing polymers, driven by the promising prospect of using the resulting materials in biomedical applications. As a result, the design of new polymeric matrices obtained from unsaturated carboxylates has become a very interesting subject adopted by many researchers. Among them, acrylates and vinylic monomers stand out, being used as building blocks for polymer synthesis, with biomedical and bioengineering applications such as ophthalmology, orthopedics, dentistry tissue engineering, and drug delivery systems [1]. For instance, polyacrylates (PAA) were used to perform drug delivery control in pH-responsive systems. Consequently, pH-sensitive hydrogels based on chitosan, cross-linking PAA to control the release of antibiotics, were prepared [2].

Poly(methyl)methacrylates (PMMA) are used as bone cement [3]. Lately, commercial formulations loaded with antibiotics have been employed to prevent/treat orthopedic infections [4]. For example, 12-methacryloyloxydodecyl-pyridinium bromide, which presents antibacterial properties, has been added to methacrylate composites and adhesives used in the dentistry area [5]. A biodegradable polymer, namely poly(propylene)fumarate (PPF), has been investigated intensively because its resulting materials are used for their regenerative medicine applications (repair of various tissues, bone tissue engineering, and hydrogel for bioprinting). The hallmarks of such materials include excellent biocompatibility, degradability, and the possibility to be tuned for time-certain resorption [6].

In addition, PMMA hydroxyapatite composites are used to produce implant materials for cranioplasty [7]. Polymeric Al_2_O_3_ nanocomposites, resulting from PMMA and poly methacrylic acid (PMAA), are reported to be denture resins with enhanced mechanical and rheological properties [8].

Also, a formulation of sodium polymethacrylate with silver nanoparticles was reported as being used for laser light-controlled drug release [9]. Moreover, the addition of silver nanoparticles to acrylic resins used in the dentistry area led to good antibacterial activity against *Escherichia coli* [1]. Furthermore, some studies succeeded in modifying conventional PMMA by the incorporation of Zr(IV) and Sn(IV) methacrylate monomers and this procedure led to high activity against biofilms produced by *Candida albicans* [10].

Considering that the incorporation of metal ions into a polymeric matrix can significantly modify its properties, some metal-containing polymers with valuable biological properties were developed [11,12,13,14]. Such materials can be obtained either by the incorporation of metal ions into a preformed polymer-bearing group with coordinative abilities, or by the polymerization or co-polymerizations of a monomeric complex possessing a ligand with a polymerizable group in the structure, for example an unsaturated carboxylate. For example, the former method was used to generate new copper (II) and nickel (II) complexes with polymeric ligands via a complex multistep protocol which starts with a methacrylate derivative. Both species displayed antimicrobial efficiency against *C. albicans*, *Escherichia coli*, and *Staphylococcus aureus*, but the Cu(II) species exhibited an enhanced potency [15].

The literature also presents metal polymer composites that result from metal acrylates and maleates, with potential use in the medicinal area [16]. Although examples of discrete complexes involved in polymerization for biomedical purposes are scarce [17], there are many studies concerning the synthesis and biological applications of several coordinative compounds bearing polymerizable groups, such as unsaturated carboxylates. This represents a suitable platform for the future development of new metal-containing polymerizable materials with valuable biological properties.

**Figure 1:**
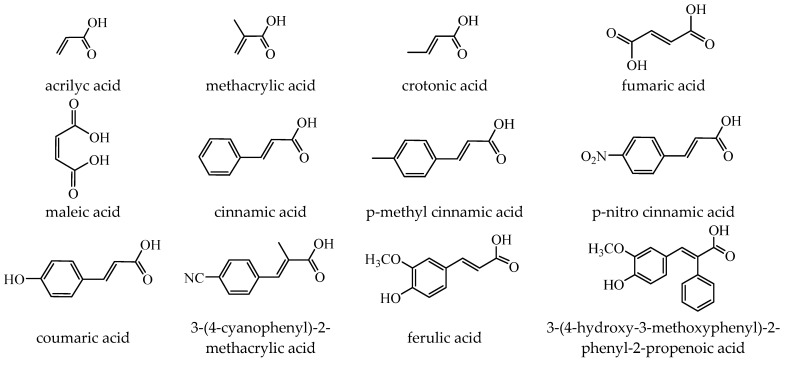
Unsaturated carboxylic acids used for obtaining monomeric coordinative compounds with biological activity.

Therefore, this paper presents aspects relating to the synthesis and biological properties of a wide range of coordinative compounds containing unsaturated carboxylates (acrylate, methacrylate, fumarate, maleate, cinnamate, ferulate, coumarate, itaconate, and their derivatives) and metal ions such as Ni(II), Cu(II), Co(II), Mn(II), Zn(II), Cd(II), Sn(IV), Pt(IV), and Ag(I). The most used unsaturated carboxylic acids for this purpose are presented in Figure 1.

In order to modulate their biological potential, most compounds also contain a N-based heterocyclic species (imidazole, pyrazole, pyridine, and quinoline derivatives) as an auxiliary ligand. As a result, the majority of these compounds are developed as antimicrobial or antitumor species, the aspects of which are detailed in the following sections.

## 2. Coordinative Compounds with Unsaturated Carboxylate Developed for Antimicrobial Applications

The continuous evolution of diseases observed in recent years requires a continuous diversification of treatment approaches.

For instance, in the case of microbial infections, both resistance [18,19,20,21,22,23,24] and biofilm (mono- and polymicrobial) development [25,26,27,28,29,30] complicate the process of finding an adequate treatment. These aspects finally produced the necessity of searching for new antimicrobials that are more efficient in both cases and the scientific results evidence that these complexes can provide promising treatment in both situations [23,25].

Consequently, the compounds described in this section are metal-containing monomers, based on acrylate, methacrylate, fumarate, maleate, cinnamate, coumarate, and itaconate as the main ligands, accompanied in some species by N-based heterocycles such as an imidazole, pyrazole, pyridine, or quinoline derivatives that exhibit good antimicrobial activity, including against resistant and biofilm-embedded strains. A selection of the most active species is presented in Table 1.

### 2.1. Coordinative Compounds with Antimicrobial Activity on Planktonic Strains

#### 2.1.1. Complexes with Acrylate and N-Based Heterocycles

A series of complexes were derived from acrylate anion. They were synthesized and characterized in order to develop species with good antimicrobial activity, both in planktonic states and biofilm-embedded ones. In order to modulate this activity, a second ligand selected from a N-based heterocycle (imidazole, benzimidazole, pyridine, and pyrazole derivatives) was used.

(a) Complexes with mixed ligands—acrylate and imidazole/alkyl-imidazole

Thus, the species [Ni(acr)_2_(Him/2-MeIm)_2_(H_2_O)_n_]∙nH_2_O **1**, **2** (Hacr = acrylic acid; Him = imidazole; *n* = 0); MeIm = methylimidazole; *n* = 1) [31] were synthesized and characterized by IR, UV-Vis-NIR, mass spectroscopy, magnetic measurements, and thermal analysis. The acrylate anions adopt a chelate coordination mode in **1** and a combination of chelate and unidentate in **2**. The antimicrobial activity was tested using an ATCC reference and clinical isolate strains, but only a moderate activity was proved for both compounds.

By using Cu(II) acrylate and several imidazole derivatives, octahedral species with *cis*- and *trans*-isomerism were obtained. These were isolated and fully characterized via single-crystal X-ray diffraction. The complexes *cis*-[Cu(acr)_2_(2-MeIm/2-EtIm)_2_]∙nH_2_O (*n* = 2) **3** and **4** (EtIm = ethylimidazole, *n* = 0) [32] were also subjected to the investigation of in vitro antibacterial activity against Gram-positive (*Enterococcus faecium*, *Bacillus subtilis, Staphylococcus aureus*) and Gram-negative (*Escherichia coli*, *Pseudomonas aeruginosa*) strains isolated from clinical samples and from deteriorated historical monuments. Both complexes exhibited improved activity in comparison with imidazole derivatives but only **4** exhibited very good activity concerning the *B. subtilis* strain, with an inhibition zone diameter (IZD) of 20 mm.

Continuing the survey, another of the reported geometric isomers formulated, *trans*-[Cu(acr)_2_(2-MeIm)_2_] **5**, presented improved activity in comparison with 2-MeIm ligand against the same bacterial strains as **3** and **4** acted on. Furthermore, similar complex with 2-ethylimidazole, *trans*-[Cu(acr)_2_(2-EtIm)_2_] **6** demonstrated higher antibacterial activity in comparison with 2-EtIm against *S. aureus* and *B. subtilis* strains. However, for *trans*-[Cu(acr)_2_(5-MeIm)_2_], **7** presented low antibacterial activity against the same strains.

**Figure 2:**
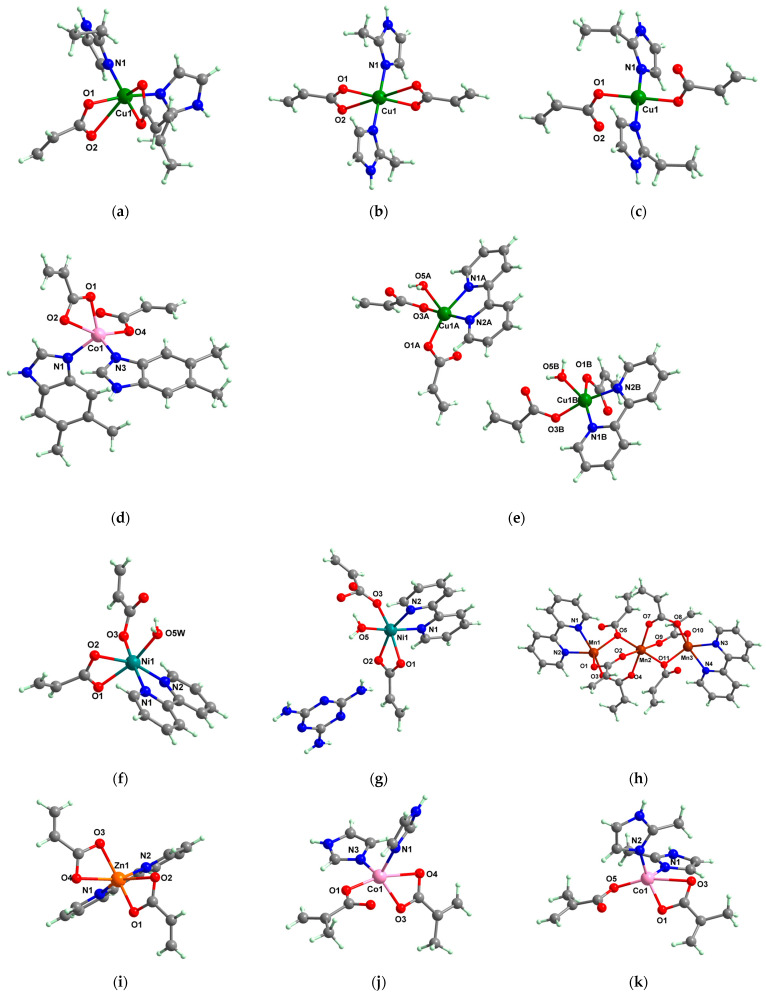

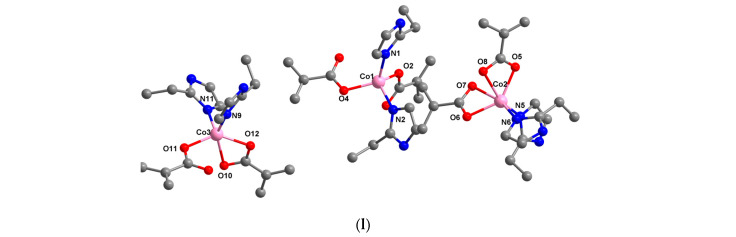
Molecular structures of *cis*-[Cu(acr)_2_(2-EtIm)_2_] **4** (**a**) [32], *trans*-[Cu(acr)_2_(2-MeIm)_2_] **5** (**b**) [32], *trans*-[Cu(acr)_2_(2-EtIm)_2_] **6** (**c**) [32], [Co(acr)_2_(5,6-Me_2_BzIm)_2_] **16** (**d**) [33], [Cu(acr)_2_(2,2′-bipy)(H_2_O)] **22** (**e**) [38], [Ni(acr)_2_(2,2′-bipy)(H_2_O)] **23** [38] (**f**), [Ni(acr)_2_(2,2′-bipy)(H_2_O)]∙MA **24** [39] (**g**), [Mn_3_(acr)_6_(2,2′-bipy)_2_] **25** [38] (**h**), [Zn(acr)_2_(2,2′-bipy)]∙H_2_O **26** [38] (**i**), [Co(Macr)_2_(HIm)_2_] **37** [43] (**j**), [Co(Macr)_2_(2-MeIm)_2_] **38** [43] (**k**), and [Co(Macr)_2_(2-EtIm)_2_] **39** [43] (**l**).

A chelate coordination mode was observed via X-ray for acrylate for complexes **4** (Figure 2a) and **5** (Figure 2b), while a unidentate mode was seen for **6** (Figure 2c). The Cu(II) adopts an octahedral distorted stereochemistry, excepting **6** where the surroundings are square–planar.

Among all the *trans*-complexes discussed above, it appears that complex **5** with 2-MeIm presents the best antimicrobial activity, evidenced by high IZD values of 35 mm *(B. subtilis)* and 30 mm (*E. faecium)*. Also, on the basis of the IZD values, it could be assumed that the antibacterial activity respects the order **5** > **6** > **7** or, considering imidazole ligands, 2-MeIm > 2-EtIm > 5-MeIm [32].

Comparing the antibacterial activity of *cis* and *trans* pair complexes, it may be stated that *trans* isomers inhibit the growth of tested bacterial strains more efficiently.

(b) Complexes with mixed ligands—acrylate and benzimidazole/alkylbenzimidazole

The violet complex [Co(acr)_2_(BzIm)_2_]∙0.5H_2_O **8** (BzIm = benzimidazole) exhibited higher antimicrobial activity, with a minimum inhibitory concentration (MIC) value of 62.5 μg∙mL^−1^ for *E. faecium* E5, *B. subtilis*, *E. coli*. and *S. aureus* [33].

The antibacterial activity of [Cu_2_(acr)_4_(BzIm)_2_] **9** was very good, with MIC values between 31.25 and 62.5 μg∙mL^−1^ against *S. aureus*, *B. subtilis*, *E. faecium* E5, and *E. coli*, the most susceptible being *S. aureus*. Instead, complex [Cu(acr)_2_(HBzIm)_2_(H_2_O)]∙(H_2_O) **10**, resulting from the same synthesis as **9** after several days of slow evaporation, exhibited a lower activity than **9 [34]**.

Continuing the inventory of benzimidazole complexes and their derivatives with biological properties, we identified species [Ni(acr)_2_(BzIm)_2_(H_2_O)]∙3H_2_O **11** and [Ni(acr)_2_(2-MeBzIm)_2_(H_2_O)]∙1.5H_2_O **12** (MeBzIm = methylbenzimidazole). These presented modest antibacterial properties, with their MIC values of 500 μg∙mL explained by a stable octahedral stereochemistry, which limits interactions with tested strains [35]. Moreover, complexes [Co(acr)_2_(2-MeBzIm)_2_]∙0.5H_2_O **13** and [Co(acr)_2_(5-MeBzIm)_2_] **14** exhibit antibacterial activity against *E. faecium* E5, *B. subtilis*, and *E. coli*, with MIC values ranging between 31.25 and 62.5 μg∙mL^−1^ [33]. Complex [Ni(acr)_2_(5-MeBzIm)_2_(H_2_O)] **15** presented rather moderate activity against *E. faecium* E5 and *E. coli* [35]. Comparing the antimicrobial activity of cobalt (II) complexes with that of nickel (II) species discussed above, it could be concluded that those of Co(II) present superior activity, this behavior being sustained by the coordinative unsaturation of this ion in the mentioned complexes.

Very good antibacterial activity was reported for [Co(acr)_2_(5,6-Me_2_BzIm)_2_] **16** (5,6-Me_2_BzIm = 5,6-dimethylbenzimidazole). This displays an interesting structure, with Co(II) configured in a distorted trigonal–bipyramidal stereochemistry and acrylate coordinated to be both unidentate and chelate (Figure 2d). This complex inhibits the growth of *E. faecium* E5, *B. subtilis*, *E. coli,* and *S. aureus*. Excepting the MIC of *E. faecium* E5, which was 62.5 μg∙mL^−1^, for all mentioned strains, the MIC values were 31.25 μg∙mL^−1^ [33].

In the same series, we obtained and characterized complexes [Cu_2_(acr)_4_(5,6-Me_2_BzIm)_2_] **17**, [Cu(acr)_2_(5,6-Me_2_BzIm)_2_(H_2_O)]∙H_2_O **18**, and [Cu(acr)_2_(5,6-Me_2_BzIm)_4_] **19**. From these species, **19** was most active against *S. aureus*, with an MIC of 250 μg∙mL^−1^ [36]. Complex [Ni(acr)_2_(5,6-Me_2_BzIm)_2_] **20** was also reported [35] to be an antibacterial agent, but its activity was rather modest.

(c) Complexes with mixed ligands—acrylate and 2,2′-bipyridine complexes

Even if antibacterial agents that contain 2,2′-bipyridine are found in the literature in great numbers, those that contain 2,2′-bipyridine and unsaturated carboxylate are limited. For instance, [Cd(acr)_2_(2,2′-bipy)]∙1.5H_2_O **21** (2,2′-bipy = 2,2′-bipyridine) was reported in [37] due to its antibacterial activity against *Shigella* sp., *Acinetobacter boumani*, *P. aeruginosa*, *S. aureus* MRSA, which was sustained by MIC values between 128 and 256 μg∙mL^−1^. In addition, the best antibacterial activity was evidenced against *Salmonella* sp.

Complexes [Cu(acr)_2_(2,2′-bipy)(H_2_O)] **22** and [Ni(acr)_2_(2,2′-bipy)(H_2_O)] **23**, tested against *S. aureus* and *E. Coli*, were found to display a modest activity [38]. Instead, [Ni(acr)_2_(2,2′-bipy)(H_2_O)]∙mlm **24** (mlm = melamine) exhibited very good activity against *S. aureus*, with an MIC value of 70 μg∙mL^−1^ [39].

The asymmetric units of **22** display two crystallographic independent entities (Figure 2e), with Cu(II) configured in a distorted octahedral stereochemistry and acrylate behaving as unidentate and chelate simultaneously [38]. The same coordination mode was evidenced for **23** (Figure 2f) and **24** (Figure 2g), along with 2,2′-bipy acting as chelate and water behaving as unidentate [38,39].

The interesting trinuclear complex [Mn_3_(acr)_6_(2,2′-bipy)_2_] **25** was reported and fully characterized through single-crystal X-ray diffraction (Figure 2h). The acrylate ions present different coordination modes such as bridges through one or two oxygen atoms, as is usually encountered in trinuclear linear complexes. These bridges link the central atom to the other two, while as a chelate bipy completes the coordination sphere for terminal Mn(II) ions. The antibacterial activity of **25** was tested against *S. aureus* and *E. coli*, when MIC values of 512 and 256 μg∙mL^−1^ were obtained. These values were much lower than those obtained for sodium acrylate [38]. Furthermore, complex [Zn(acr)_2_(2,2′-bipy)]∙H_2_O **26** was tested against the same bacterial strains as **25**, and the result was that it was more active against *S. aureus*, a fact proven by an MIC value of 128 μg∙mL^−1^ [38].

The complex [Cd(acr)_2_(phen)(H_2_O)] **27** (phen = 1,10-phenantroline) was found to be active against *Acinetobacter boumani*, with an MIC of 64 μg∙mL^−1^, and against *P. aeruginosa* and *S. aureus* MRSA strains, with MICs of 256 μg∙mL^−1^ for both strains [37].

(d) Complexes with mixed ligands—acrylate and pyrazole/pyrazole derivatives

Complexes with mixed ligands, dubbed [Co(acr)_2_(Hpz)_2_] **28** (Hpz = 1H-pyrazole), [Co(acr)_2_(3-MeHpz)_2_] **29** (MeHpz = methyl-1H-pyrazole), [Co(acr)_2_(4-Me-Hpz)_2_] **30**, and [Co(acr)_2_(dmpz)_2_] **31** (dmpz = 3,5-dimethyl-1H-pyrazole), were tested against *S. aureus* 1263 MRSA, *E. coli*, *K. preumoniae*, and *B. subtilis*, but good activity was only exhibited against *B. subtilis*, with an MIC of 125 μg∙mL^−1^ [40].

#### 2.1.2. Complexes with Methacrylate/Methacrylate Derivatives and Different N-Donor Ligands

Even though methacrylate/methacrylate-based products are used for different dental and medical applications [41], complexes with mixed ligands, methacrylate, and different accompanying N-donor heterocycles with biological properties are scarcely reported in the literature. For example, series of such species were reported, with a general formula of [M(macr)_2_(4,4′-bipy)]∙nH_2_O (Hmacr = methacrylic acid; 4,4′-bipy = 4,4′-bipyridine). Among these, it is worth mentioning that [Co(macr)_2_(4,4′-bipy)]∙0.5H_2_O **32**, [Cu(macr)_2_(4,4′-bipy)]∙0.5H_2_O **33**, [Mn(macr)_2_(4,4′-bipy)] **34**, and [Ni(macr)_2_(4,4′-bipy)]∙1.5H_2_O **35** presented very good activity against the resistant strain of *E. coli* ESBL 1576, with MIC values of 31.25 and 62.5 μg∙mL^−1^ [42].

From other series, [Co(macr)_2_(HIm)_2_] **37**, [Co(macr)_2_(2-MeIm)_2_] **38** and [Co(macr)_2_(2-EtIm)_2_] **39** were reported as being good antibacterial agents. The structures of these compounds can be observed in Figure 2j–l. For instance, complex **37** presents very good antibacterial activity against *E. coli* (MIC 31.2 μg∙mL^−1^), *S. aureus* (MIC 15.6 μg∙mL^−1^), and *E. faecalis* (MIC 31.2 μg∙mL^−1^), while **38** is active against *E. faecalis* (MIC 62.5 μg∙mL^−1^), *E. coli* (MIC = 31.2 μg∙mL^−1^), *P. aeruginosa* (MIC = 31.25 μg∙mL^−1^), and *S. aureus* (MIC = 15.6 μg∙mL^−1^), respectively. Concluding, the Gram-positive strains were significantly more sensitive to the investigated complexes, in comparison to the ligands. An interesting structural feature was observed for complex **39**, with the asymmetric unit containing three crystalographically independent molecules, where the metallic ions adopted different stereochemistries (distorted octahedral, tetrahedral, and distorted square–pyramidal) (Figure 2l) [43].

**Figure 3:**
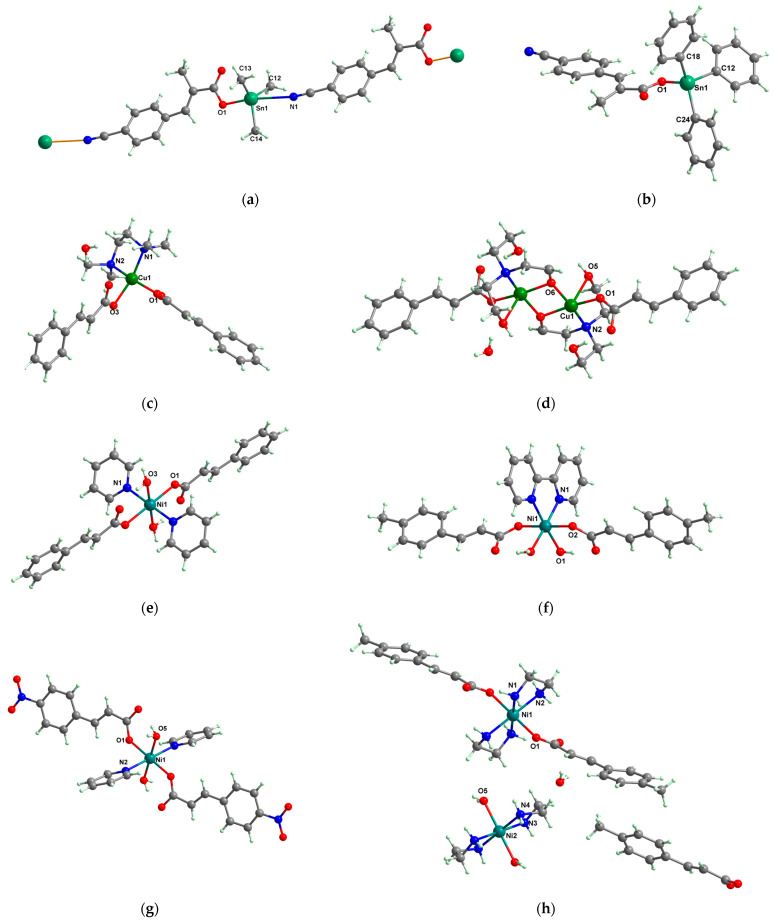
Molecular structures of [Me_3_Sn(cpma)] **43** (**a**) [44], [Ph_3_Sn(cpma)] **45** (**b**) [44], [Cu(cin)_2_(tmeda)]∙0.7H_2_O **46** (**c**) [47], [Cu_2_(cin)_2_(tea)](H_2_O) **47** (**d**) [48], *trans*-[Ni(cin)_2_(py)_2_(H_2_O)_2_] **48** (**e**) [49], *trans,cis*-[Ni(mcin)_2_(2,2′-bipy)(H_2_O)_2_] **49** [49] (**f**), *trans*-[Ni(ncin)_2_(py)_2_(H_2_O)_2_] **51** [49] (**g**), and {*trans*-[Ni(mcin)_2_(en)_2_][Ni(en)_2_(H_2_O)_2_](mcin)∙H_2_O} **50** [49] (**h**).

Antibacterial properties were found for a series of organotin compounds with 3-(4-cyanophenyl)-2-methylacrylate (Hcpma); these were formulated as [Me_2_Sn(cpma)_2_] **40**, [Bu_2_Sn(cpma)_2_] **41**, [Oct_2_Sn(cpma)_2_] **42**, [Me_3_Sn(cpma)] **43** (Figure 3a)**,** [Bu_3_Sn(cpma)] **44**, and [Ph_3_Sn(cpma)] **45** (Figure 3b). All complexes were mononuclear, with carboxylate acting in a unidentate manner, excepting **43**, for which the single-crystal X-ray diffraction evidenced a polymeric structure with carboxylate as the bridge. Compound **45** presented the best antibacterial activity against *S. aureus* strain (IZD = 30 mm), followed by **44** (IZD = 22 mm). Complex **43** was the most active against *Bortedella bronchiseptica* (IZD = 30 mm), and we found that compound **45** had good activity against *Micrococcus luteus* with an IZD of 27 mm. Even if all species from this series showed antibacterial abilities against the tested strains, the best antibacterial activity was shown by **43** and **45** [44].

#### 2.1.3. Complexes with Cinnamate/Cinnamate Derivatives and Different N-Donor Ligands

Cinnamic acid and its derivatives are encountered in plants and are reported due to their antibacterial, antifungal, anticancer, antiparasitic properties, and potential therapeutic behavior against Alzheimers’ disease [45,46]. Given these attributes, it was expected that cinnamate-based complexes would exhibit similar properties, and so the literature was accordingly scanned to identify such complexes.

A cinnamate complex, [Cu(cin)_2_(tmeda)]∙0.7H_2_O **46** (Hcin = cinnamic acid; tmeda = *N,N,N’,N’-*tetramethylenediamine), was reported due to its remarkable antibacterial activity against *Bacillus spizizenii* (MIC 10 μg∙mL^−1^) and *Staphylococcus aureus* (MIC 25 μg∙mL^−1^). In addition, moderate activity was determined against *Enterobacter aerogenes*, E. coli, K. pneumoniae, and *P. aeruginosa*. Copper(II) adopts a distorted octahedral stereochemistry, with both cinnamate and amine ligands acting as chelates (Figure 3c) [47].

Complex [Cu_2_(tea)_2_(cin)_2_](H_2_O) **47** (Htea = triethanolamine) was synthesized and fully characterized as a binuclear species through single-crystal X-ray diffraction. The octahedral stereochemistry of Cu(II) ions was assured by the unidentate cinnamate and triethanolamine, acting both as a tetradentate chelate and a bridge through the deprotonated hydroxyl group (Figure 3d). The complex exhibited very good inhibitory activity against an *S. aureus* strain in a planktonic state with an MIC of 25 μg∙mL^−1^, showing activity comparable with that of cephalexin and its complexes [48].

Several Ni(II) complexes with cinnamate derivatives, namely, [Ni(cin)_2_(py)_2_(H_2_O)_2_] **48** (py = pyridine), [Ni(mcin)_2_(2,2′-bipy)(H_2_O)_2_] **49** (Hmcin = p-methyl cinnamic acid), {[Ni(mcin)_2_(en)_2_][Ni(en)_2_(H_2_O)_2_](mcin)∙H_2_O} **50** (en = ethylenediamine), and [Ni(ncin)_2_(py)_2_(H_2_O)_2_] **51** (Hncin = p-nitro cinnamic acid), were obtained and characterized as mononuclear species, with cinnamate derivatives acting in a unidentate manner (Figure 3d–h). Complexes **48**, **50**, and **51** presented significant activity against *Micrococcus luteus,* with IZDs of about 20 mm [49].

A derivative of cinnamic acid, namely, p-coumaric acid (p-hydroxicinnamic acid), is widely encountered in nature, its antibacterial properties being intensively studied [50,51,52]. Although the antibacterial properties of this compound are fully proven, reports of complexes with such properties are scarce.

For example, a polynuclear complex that was formulated [Zn_4_(Hcou)_8_(H_2_O)_6_]∙4H_2_O **52** (Hcou = coumaric acid) has been reported due to its antibacterial properties, evaluated on the basis of percentage growth inhibition (PGI). More specifically, it was found that complex **52** presents significant activity against *S. aureus*, with a PGI of 77 [53].

Furthermore, ferulic acid, also found in the vegetal species, has proven its antimicrobial properties [52]. Considering these, complex [Zn(Hfer)_2_]*·*1.5H_2_O **53** (H_2_fer = ferulic acid or 3-methoxy-4-hydroxicinnamic acid) revealed significant activity against *E. coli* (PGI 97.1%), *B. subtilis* (PGI 90.8%), *S. aureus* (PGI 93.7%), and *P. vulgaris* (PGI 96.5%), with a level much higher than that of ferulic acid or sodium ferulate [54].

Organotin (IV) compounds receive special attention due to their antibacterial activities and their use as biocidal agents [55,56,57]. Considering these aspects, [Me_3_Sn(hmpp)] **54** (Me = methyl; Hhmpp = 3-(4-hydroxy-3-methoxyphenyl)-2-phenylpropenoic acid) was tested and presented a good and enhanced activity against *P. aeruginosa* (IZD 18 mm) in comparison with a ligand [58].

The [Eu(cfa)_3_(H_2_O)_3_]·2H_2_O **55** (Hcfa = caffeic acid) also exhibits an enhanced activity against *E. coli* and *B. subtilis* compared to Hcfa, behavior which can be explained by increased lipophilicity. However, the activity was modest, with an MIC of 400 μg∙mL^−1^ [59].

**Table 1:** Coordination compounds with unsaturated carboxylates with antimicrobial activity.

Compound Formulation	Unsaturated Carboxylate Ligand	Auxiliary Ligand	Biological Activity	Ref.
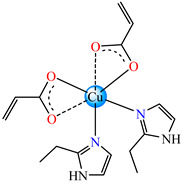 *cis*-[Cu(acr)_2_(2-EtIm)_2_] **4**	acrylate (chelate) *	2-ethylimidazole	**ABA** **: *B. subtilis* (IZD = 22 mm)	[32]
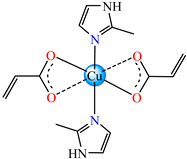 *trans*-[Cu(acr)_2_(2-MeIm)_2_] **5**	acrylate (chelate) *	2-methylimidazole	**ABA**: *E. faecium* (IZD = 30 mm), *B. subtilis* (IZD = 35 mm), *S. aureus* (IZD = 18 mm)	[32]
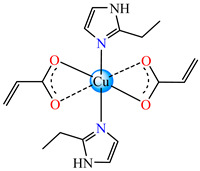 *trans*-[Cu(acr)_2_(2-EtIm)_2_] **6**	acrylate (unidentate semicoordination) *	2-ethylimidazole	**ABA**: *B. subtilis* (IZD = 27 mm) *P. aeruginosa* (IZD = 18 mm)	[32]
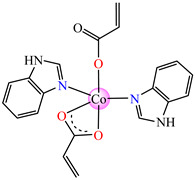 [Co(acr)_2_(HBzIm)_2_]∙0.5H_2_O **8**	acrylate	benzimidazole	**ABA**: *E. faecium* E5 (MIC = 62.5 μg∙mL^−1^), *B. subtilis* ATCC 6683 (MIC = 62.5 μg∙mL^−1^), *E. coli* ATCC 25922 (MIC = 62.5 μg∙mL^−1^), *S. aureus* (MIC = 62.5 μg∙mL^−1^) **AFA** ***: *C. albicans* (MIC = 62.5 μg∙mL^−1^)	[33]
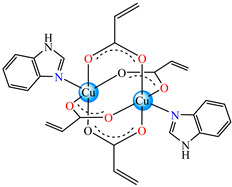 [Cu_2_(acr)_4_(HBzIm)_2_] **9**	acrylate	benzimidazole	**ABA**: *S. aureus* ATCC 6538 (MIC = 31.25 μg∙mL^−1^), *B. subtilis* 6683 (MIC = 62.5 μg∙mL^−1^), *E. faecium* E5 (MIC = 62.5 μg∙mL^−1^), *E. coli* ATCC 25922 (MIC = 62.5 μg∙mL^−1^) **AFA**: *C. albicans* 1760 (MIC = 62.5 μg∙mL^−1^)	[34]
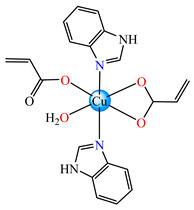 [Cu(acr)_2_(HBzIm)_2_(H_2_O)]∙(H_2_O) **10**	acrylate	benzimidazole	**ABA**: *S. aureus* ATCC 6538 (MIC = 62.5 μg∙mL^−1^), *B. subtilis* 6683 (MIC = 125 μg∙mL^−1^), *E. faecium* E5 (MIC = 62.5 μg∙mL^−1^), *E.coli* ATCC 25922 (MIC = 125 μg∙mL^−1^)	[34]
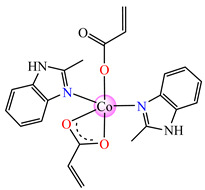 [Co(acr)_2_(2-MeBzIm)_2_]∙0.5H_2_O **13**	acrylate	2-methylbenzimidazole	**ABA**: *E. faecium* E5 (MIC = 62.5 μg∙mL^−1^), *B. subtilis* ATCC 6683 (MIC = 31.25 μg∙mL^−1^), *S. aureus* (MIC = 31.25 μg∙mL^−1^) *E. coli* ATCC 25922 (MIC = 31.25 μg∙mL^−1^) **AFA**: *C. albicans* (MIC = 62.5 μg∙mL^−1^)	[33]
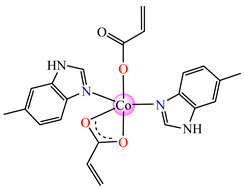 [Co(acr)_2_(5-MeBzIm)_2_] **14**	acrylate	5-methylbenzimidazole	**ABA**: *E. faecium* E5 (MIC = 62.5 μg∙mL^−1^), *B. subtilis* ATCC 6683 (MIC = 62.5 μg∙mL^−1^), *S. aureus* (MIC = 31.25 μg∙mL^−1^), *E. coli* ATCC 25922 (MIC = 62.5 μg∙mL^−1^) **AFA**: *C. albicans* (MIC = 62.5 μg∙mL^−1^)	[33]
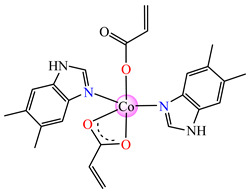 [Co(acr)_2_(5,6-Me_2_BzIm)_2_] **16**	acrylate (unidentate + chelate) *	5,6-dimethylbenzimidazole	**ABA**: *E. faecium* E5 (MIC = 62.5 μg∙mL^−1^), *B. subtilis* ATCC 6683 (MIC = 31.25 μg∙mL^−1^), *E. coli* ATCC 25922 (MIC = 31.25 μg∙mL^−1^), *S. aureus* (MIC = 31.25 μg∙mL^−1^) **AFA**: *C. albicans* (MIC = 31.25 μg∙mL^−1^)	[33]
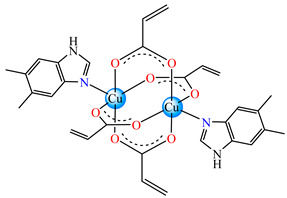 [Cu_2_(acr)_4_(5,6-Me_2_BzIm)_2_] **17**	acrylate	5,6-dimethylbenzimidazole	**ABA**: MRSA 1263 (MIC = 250 μg∙mL^−1^)	[36]
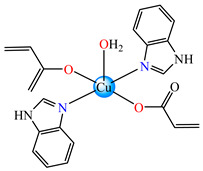 [Cu(acr)_2_(5,6-Me_2_BzIm)_2_(H_2_O)]∙H_2_O **18**	acrylate	5,6-dimethylbenzimidazole	**ABA**: *E. coli* (MIC = 125 μg∙mL^−1^)*, K. pneumoniae* (MIC = 125 μg∙mL^−1^)*,* MRSA 1263 (MIC = 125 μg∙mL^−1^)*, B. subtilis* (MIC = 125 μg∙mL^−1^)	[36]
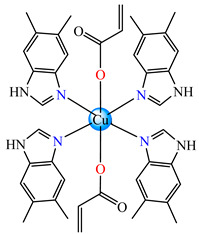 [Cu(acr)_2_(5,6-Me_2_BzIm)_4_] **19**	acrylate	5,6-dimethylbenzimidazole	**ABA**: *S. aureus* (MIC = 250 μg∙mL^−1^)*,* MRSA 1263 (MIC = 250 μg∙mL^−1^)	[36]
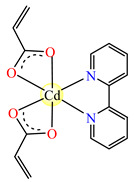 [Cd(acr)_2_(2,2′-bipy)]∙1.5H_2_O **21**	acrylate	2,2′-bipyridine	**ABA**: *Shigella* sp. (MIC = 256 μg∙mL^−1^), *Acinetobacter boumani* (MIC = 128 μg∙mL^−1^), *P. aeruginosa* 1700 (MIC = 256 μg∙mL^−1^), *S. aureus* MRSA (MIC = 256 μg∙mL^−1^) **AFA**: *C. albicans* (MIC = 256 μg∙mL^−1^)	[37]
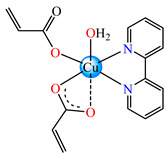 [Cu(acr)_2_(2,2′-bipy)(H_2_O)] **22**	acrylate (unidentate) *	2,2′-bipyridine	**ABA**: *E. coli* (MIC = 128 μg∙mL^−1^) **AFA**: *C. albicans* (MIC = 128 μg∙mL^−1^)	[38]
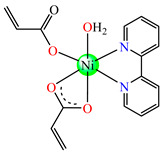 [Ni(acr)_2_(2,2′-bipy)(H_2_O)] **23**	acrylate *(unidentate + chelate)*	2,2′-bipyridine	**AFA**: *C. albicans* (MIC = 128 μg∙mL^−1^)	[38]
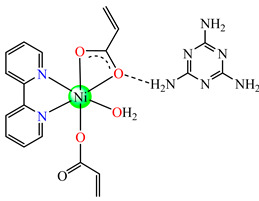 [Ni(acr)_2_(2,2′-bipy)(H_2_O)]∙MA **24**	acrylate *(unidentate + chelate)*	2,2′-bipyridine	**ABA**: *S. aureus* ATCC 25923 (MIC = 70 μg∙mL^−1^)	[39]
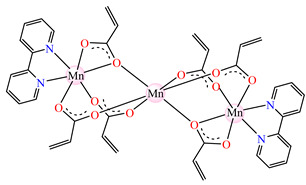 [Mn_3_(acr)_6_(2,2′-bipy)_2_] **25**	acrylate (bridge through one or two oxygen atoms) *	2,2′-bipyridine	**ABA**: *E. coli* (MIC = 256 μg∙mL^−1^) **AFA**: *C. albicans* (MIC = 128 μg∙mL^−1^)	[38]
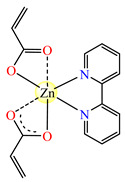 [Zn(acr)_2_(2,2′-bipy)]∙H_2_O **26**	acrylate (chelate) *	2,2′-bipyridine	**ABA**: *S. aureus* (MIC = 128 μg∙mL^−1^) **AFA**: *C. albicans* (MIC = 128 μg∙mL^−1^)	[38]
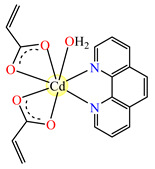 [Cd(acr)_2_(phen) (H_2_O)] **27**	acrylate	1,10-phenantroline	**ABA**: *Acinetobacter boumani* (MIC = 64 μg∙mL^−1^), *P. aeruginosa* 1700 (MIC = 256 μg∙mL^−1^), *S. aureus* MRSA (MIC = 256 μg∙mL^−1^) **AFA**: *C. albicans* (MIC = 256 μg∙mL^−1^)	[37]
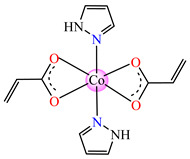 [Co(acr)_2_(Hpz)_2_] **28**	acrylate	1H-pyrazole	**ABA**: *B. subtilis* (MIC = 125 μg∙mL^−1^)	[40]
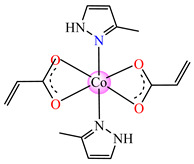 [Co(acr)_2_(3-MeHpz)_2_] **29**	acrylate	3-methyl-1H-pyrazole	**ABA**: *B. subtilis* (MIC = 125 μg∙mL^−1^)	[40]
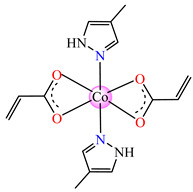 [Co(acr)_2_(4-MeHpz)_2_] **30**	acrylate	4-methyl-1H-pyrazole	**ABA**: *B. subtilis* (MIC = 125 μg∙mL^−1^)	[40]
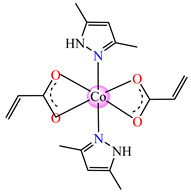 [Co(acr)_2_(dmpz)_2_] **31**	acrylate	3,5-dimethyl-1H-pyrazole	**ABA**: *B. subtilis* (MIC = 125 μg∙mL^−1^)	[40]
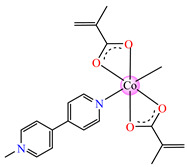 [Co(Macr)_2_(4,4′-bipy)]∙0.5H_2_O **32**	methacrylate	4,4′-bipyridine	**ABA**: *S. aureus* (MIC = 125 μg∙mL^−1^)*, P. aeruginosa* (MIC = 125 μg∙mL^−1^)*, E. coli* ESBL 1576 (MIC = 31.25 μg∙mL^−1^), *E. coli* ATCC 25922 (MIC = 31.25 μg∙mL^−1^)	[42]
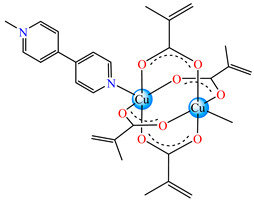 [Cu(Macr)_2_(4,4′-bipy)]∙0.5H_2_O **33**	methacrylate	4,4′-bipyridine	**ABA**: *P. aeruginosa* (MIC = 125 μg∙mL^−1^)*, E. coli* ESBL 1576 (MIC = 62.5 μg∙mL^−1^), *E. coli* ATCC 25922 (MIC = 62.5 μg∙mL^−1^)	[42]
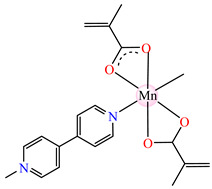 [Mn(Macr)_2_(4,4′-bipy)] **34**	methacrylate	4,4′-bipyridine	**ABA**: *S. aureus* (MIC = 125 μg∙mL^−1^)*, P. aeruginosa* (MIC = 125 μg∙mL^−1^)*, E. cloacae* (MIC = 250 μg∙mL^−1^), *E. coli* ESBL 1576 (MIC = 31.25 μg∙mL^−1^), *E. coli* ATCC 25922 (MIC = 62.5 μg∙mL^−1^)	[42]
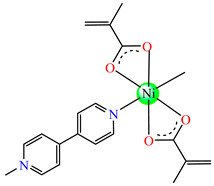 [Ni(Macr)_2_(4,4′-bipy)]∙1.5H_2_O **35**	methacrylate	4,4′-bipyridine	**ABA**: *S. aureus* (MIC = 31.25 μg∙mL^−1^)*, P. aeruginosa* (MIC = 250 μg∙mL^−1^)*, E. coli* ESBL 1576 (MIC = 31.25 μg∙mL^−1^), *E. coli* ATCC 25922 (MIC = 62.5 μg∙mL^−1^)	[42]
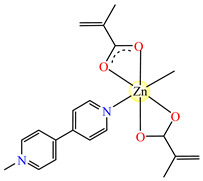 [Zn(Macr)_2_(4,4′-bipy)]∙0.5H_2_O **36**	methacrylate	4,4′-bipyridine	**ABA**: *S. aureus* (MIC = 125 μg∙mL^−1^)*, P. aeruginosa* (MIC = 125 μg∙mL^−1^)*, E. cloacae* (MIC = 250 μg∙mL^−1^), *E. coli* ESBL 1576 (MIC = 31.25 μg∙mL^−1^), *E. coli* ATCC 25922 (MIC = 62.5 μg∙mL^−1^)	[42]
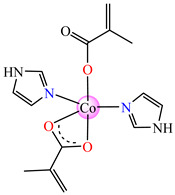 [Co(Macr)_2_(HIm)_2_] **37**	methacrylate (unidentate + chelate) *	imidazole	**ABA**: *E. coli* ATCC 8739 (MIC = 31.2 μg∙mL^−1^), *P. aeruginosa* ATCC 1671 (MIC = 62.5 μg∙mL^−1^), *S. aureus* ATCC 6538 (MIC = 15.6 μg∙mL^−1^), *E. faecalis* ATCC 29212 (MIC = 31.2 μg∙mL^−1^) **AFA**: *C. albicans* ATCC 26790 (MIC = 7.8 μg∙mL^−1^)	[43]
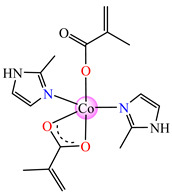 [Co(Macr)_2_(2-MeIm)_2_] **38**	methacrylate (unidentate + chelate) *	2-methylimidazole	**ABA**: *E. coli* ATCC 8739 (MIC = 31.2 μg∙mL^−1^), *P. aeruginosa* ATCC 1671 (MIC = 15.6 μg∙mL^−1^), *S. aureus* ATCC 6538 (MIC = 15.6 μg∙mL^−1^), *E. faecalis* ATCC 29212 (MIC = 31.2 μg∙mL^−1^)	[43]
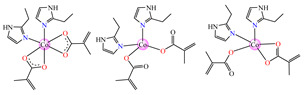 [Co(Macr)_2_(2-EtIm)_2_] **39**	methacrylate (unidentate + chelate in left unit; unidentate in middle unit; chelate in right unit) *	2-ethylimidazole	**ABA**: *E. coli* ATCC 8739 (MIC = 125 μg∙mL^−1^), *P. aeruginosa* ATCC 1671 (MIC = 31.2 μg∙mL^−1^), *S. aureus* ATCC 6538 (MIC = 15.6 μg∙mL^−1^), *E. faecalis* ATCC 29212 (MIC = 62.5 μg∙mL^−1^) **AFA**: *C. albicans* ATCC 26790 (MIC = 15.6 μg∙mL^−1^)	[43]
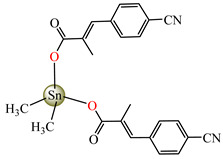 [Me_2_Sn(cpma)_2_] **40**	3-(4-cyanophenyl)-2-methylacrylate	methyl	**ABA**: *S. aureus* (IZD = 20 mm), *E. coli* (IZD = 20 mm), *Bortedella bronchiseptica* (IZD = 25 mm), *Micrococcus luteus* (IZD = 20 mm) **AFA**: *A. fumigatus* (PGI = 65%)	[44]
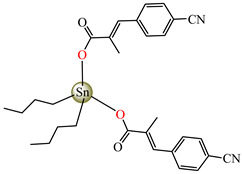 [Bu_2_Sn(cpma)_2_] **41**	3-(4-cyanophenyl)-2-methylacrylate	n-butane	**ABA**: *S. aureus* (IZD = 20 mm), *E. coli* (IZD = 20 mm), *Bortedella bronchiseptica* (IZD = 25 mm) **AFA**: *A. flavus* (PGI = 50%), *A. fumigatus* (PGI = 55%), *Fusarium solani* (PGI = 95%)	[44]
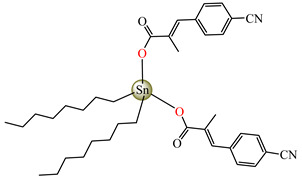 [Oct_2_Sn(cpma)_2_] **42**	3-(4-cyanophenyl)-2-methylacrylate	n-octane	**ABA**: *E. coli* (IZD = 16 mm), *Bortedella bronchiseptica* (IZD = 10 mm)	[44]
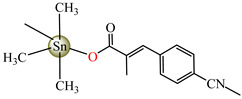 [Me_3_Sn(cpma)]_n_**43**	3-(4-cyanophenyl)-2-methylacrylate (unidentate) *	methyl	**ABA**: *S. aureus* (IZD = 20 mm), *E.coli* (IZD = 25 mm), *Bortedella bronchiseptica* (IZD = 30 mm) **AFA**: *A. flavus* (PGI = 96%), *A. niger* (PGI = 100%), *A. fumigatus* (PGI = 100%), *Fusarium solani* (PGI = 100%)	[44]
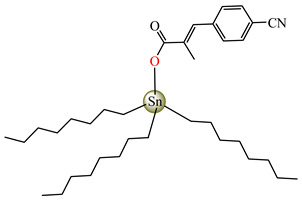 [Bu_3_Sn(cpma)] **44**	3-(4-cyanophenyl)-2-methylacrylate	butyl	**ABA**: *S. aureus* (IZD = 22 mm), *E. coli* (IZD = 23 mm), *Bortedella bronchiseptica* (IZD = 25 mm), *Micrococcus luteus* (IZD = 20 mm) **AFA**: *A. niger* (PGI = 58%), *A. fumigatus* (PGI = 75%), *Fusarium solani* (PGI = 75%)	[44]
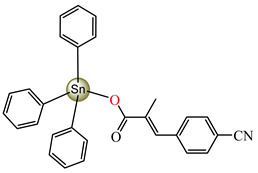 [Ph_3_Sn(cpma)] **45**	3-(4-cyanophenyl)-2-methylacrylate (bidentate chelate) *	phenyl	**ABA**: *S. aureus* (IZD = 30 mm), *E. coli* (IZD = 20 mm), *Bortedella bronchiseptica* (IZD = 20 mm), *Micrococcus luteus* (IZD = 27 mm) **AFA**: *A. flavus* (PGI = 98%), *A. niger* (PGI = 97%), *A. fumigatus* (PGI = 100%), *Fusarium solani* (PGI = 96%)	[44]
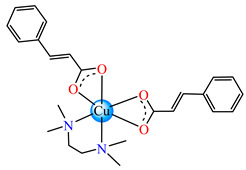 [Cu(cin)_2_(tmeda)]∙0.7H_2_O **46**	cinnamate (unidentate) *	*N,N,N’,N’*-tetramethylenediamine	**ABA**: *Bacillus spizizenii* (MIC = 10 μg∙mL^−1^), *S. aureus* (MIC = 25 μg∙mL^−1^)	[47]
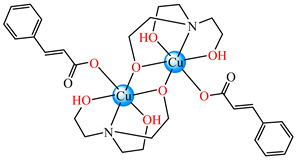 [Cu_2_(cin)_2_(tea)](H_2_O) **47**	cinnamate (unidentate) *	triethanolamine	**ABA**: *S. aureus* (MIC = 25 μg∙mL^−1^)	[48]
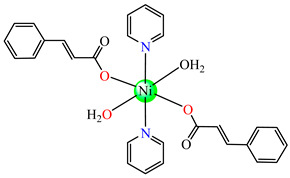 *trans*-[Ni(cin)_2_(py)_2_(H_2_O)_2_] **48**	cinnamate (unidentate) *	pyridine	**ABA**: *Micrococcus luteus* (IZD = 25 mm)	[49]
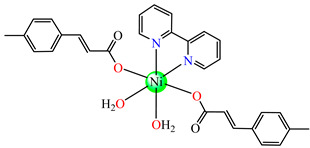 *trans,cis*-[Ni(mcin)_2_(2,2′-bipy)(H_2_O)_2_] **49**	p-methylcinnamate (unidentate) *	2,2′-bipyridine	**ABA**: *S. aureus* (IZD = 20 mm)	[49]
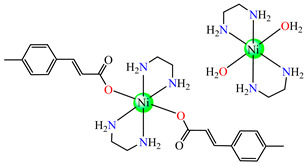 {*trans*-[Ni(mcin)_2_(en)_2_][Ni(en)_2_(H_2_O)_2_](mcin)∙H_2_O} **50**	p-methylcinnamate (unidentate) *	ethylenediamine	**ABA**: *Micrococcus luteus* (IZD = 21 mm)	[49]
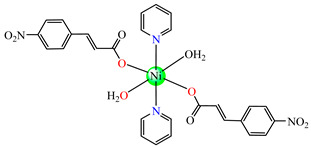 *trans*-[Ni(ncin)_2_(py)_2_(H_2_O)_2_] **51**	p-nitrocinnamate (unidentate) *	pyridine	**ABA**: *Micrococcus luteus* (IZD = 21 mm), *B. subtilis* (IZD = 18 mm)	[49]
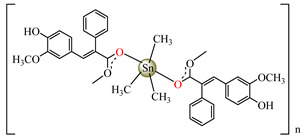 [Me_3_Sn(hmpp)_2_]_n_**54**	3-(4-hydroxy-3-methoxyphenyl)-2-phenylpropenoate	methyl	**ABA**: *E. coli* (IZD = 15 mm), *B. subtilis* (IZD = 15 mm), *P. aeruginosa* (IZD = 18 mm)	[58]
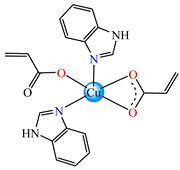 [Cu(acr)_2_(HBzIm)_2_] **60**	acrylate	benzimidazole	**AFA**: *C. albicans* 1760 (MIC = 31.25 μg∙mL^−1^)	[34]
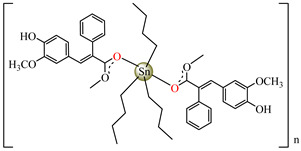 [Bu_3_Sn(hmpp)] **61**	3-(4-hydroxy-3-methoxyphenyl)-2-phenylpropenoate	butyl	**AFA**: *A. flavus* (PGI = 70%), *Microsporum canis* (PGI = 65%)	[58]
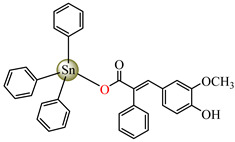 [Ph_3_Sn(hmpp)] **62**	3-(4-hydroxy-3-methoxyphenyl)-2-phenylpropenoate	phenyl	**AFA**: *Microsporum canis* (PGI = 60%)	[58]

* carboxylate coordination mode evidenced in X-ray structure; ** ABA = antibacterial activity; *** AFA = antifungal activity.

#### 2.1.4. Complexes with Maleate and Different Heterocyclic Amine

Maleic acid is seldom encountered as a ligand in complexes that are mainly discussed from a structural perspective [60,61,62] and is rarely associated with biological properties, its antimicrobial activity against *E. faecalis* biofilm being reported on instead [63].

For example, a series of Co(II) complexes with maleate and heterocyclic derivatives was evaluated to assess their antibacterial properties. Consequently, complexes [Co(mal)(2-apy)] **56** (H_2_mal = maleic acid; 2-apy = 2-aminopyridine), [Co(mal)(qn)_2_] **57** (qn = quinoline), [Co(mal)(iqn)_2_] **58** (iqn = iso-quinoline) and K[Co(mal)(8-hqn)] **59** (hqn = hydroxiquinoline) were tested on several pathogenic bacteria and the results were more than satisfactory. More specifically, complexes **57**, **58**, and **59** inhibited the growth of *Streptococcus haemolyticus* efficiently, with IZD values of 32, 33, and 37 mm, respectively. Among these, **59** presented the best antimicrobial activity against all tested bacterial strains, the highest IZD of 40 mm being found for *Shigella dysenteriae* [64].

### 2.2. Coordinative Compounds with Unsaturated Carboxylate with Activity on Bacterial Biofilm

Bacterial biofilms are usually developed on both living or abiotic surfaces, thus generating chronic and persistent infections that cannot be eradicated with classical antimicrobials due to their increased tolerance to antimicrobials and the host’s immune system. Fortunately, several complexes based mostly on 3d ions have shown promising potential for fighting biofilm-associated infections as a result of their large spectrum of antibacterial biofilm activities [23,25]. Some species from among them, bearing unsaturated carboxylate, are presented below.

Hence, the complexes [Ni(acr)_2_(HBzIm)_2_(H_2_O)]∙3H_2_O **11**, [Ni(acr)_2_(5-MeBzIm)_2_(H_2_O)] **15**, [Ni(acr)_2_(5,6-Me_2_BzIm)_2_] **20** exhibited an inhibitory effect upon microbial adherence to an inert substrate against *E. faecium* E5, with a minimum biofilm eradication concentration (MBEC) of 250 μg∙mL^−1^ [35]. In addition, related complex [Ni(acr)_2_(2-MeBzIm)_2_(H_2_O)]∙1.5H_2_O **12** [35] evidenced activity against *Klebsiella pneumoniae* at a MBEC of 250 μg∙mL^−1^.

Furthermore, complexes [Cu_2_(acr)_4_(5,6-Me_2_BzIm)_2_] **17**, [Cu(acr)_2_(5,6-Me_2_BzIm)_2_(H_2_O)]∙H_2_O **18**, and [Cu(acr)_2_(5,6-Me_2_BzIm)_4_] **19** were subjected to investigations to find out how they interact with biofilms formed by different bacterial strains. It was evidenced that the effect was either inhibitory or stimulatory, depending on the tested strain or the concentration of the tested complex [36]. It was found that complexes **17** and **18** presented inhibitory effects upon adherence of *K. pneumoniae* 1204 (1.95 μg∙mL^−1^) and *C. albicans* (1.95 μg∙mL^−1^), respectively. Also, complex **19** presented the most evident inhibitory effect upon the adherence ability of *E. coli* 13147 and *K. preumoniae* 1204, even at a subinhibitory concentration (1.95 μg∙mL^−1^). The antibiofilm activity of **19** in the case of *E. coli* 13529 and *C. albicans* 249 was only evidenced above concentrations of 7.81 and 125 μg∙mL^−1^, respectively.

The co-crystal [Ni(acr)_2_(2,2′-bipy)(H_2_O)]∙mlm **24** [39] was investigated against *S. aureus* 25923, *E. faecalis* 29212, *E. coli* 25922, and *P. aeruginosa* 27853, with a resulting MBEC range between 150 and 1250 μg∙mL^−1^, very close to the MIC values presented in Section 2.1. Co-crystal **24** presents a better antibiofilm effect by comparison to melamine and is more active on Gram-positive strains.

The complexes formulated with methacrylate and imidazole/imidazole derivatives, namely, [Co(macr)_2_(HIm)_2_] **37**, [Co(macr)_2_(2-MeIm)_2_] **38**, and [Co(macr)_2_(2-EtIm)_2_] **39,** proved their efficacy against biofilms developed by *E. coli*, *P. aeruginosa*, *S. aureus*, and *E. faecalis*, with MBEC values ranging between 15.6 and 62.5 μg∙mL^−1^. Among the tested bacterial strains, the most susceptible was *S. aureus* with an MBEC value of 15.6 μg∙mL^−1^, followed closely by *P. aeruginosa*, with MBEC values that range between 15.6 and 31.2 μg∙mL^−1^. In the crystal structures of the neutral species **37** and **38**, two unidentate imidazole and two methacrylate ions can be observed. These are both unidentate and chelate, existing in a trigonal–bipyramidal stereochemistry (Figure 2j,k). The structure of complex **39** reveals very interesting features, with three crystallographically independent neutral molecules in the asymmetric unit (Figure 2l). All three Co(II) ions exhibit different stereochemistries: Co1 presents a square–pyramidal geometry, Co2 presents a tetrahedral stereochemistry, while Co3 adopts a slightly distorted octahedral one (Table 1). Furthermore, the methacrylate acts either as chelate, unidentate, or in both fashions for the three independent Co(II) moieties [43].

### 2.3. Coordinative Compounds with Unsaturated Carboxylate with Antifungal Properties

The geometric isomers *cis*-[Cu(acr)_2_(2-MeIm)_2_]∙2H_2_O **3** and *trans*-[Cu(acr)_2_(2-MeIm)_2_] **5**, which exhibit antibacterial activity (see Section 2.1), were also tested on antifungal strains *C. albicans*, *Penicillium* sp., and *Aspergillus* sp., and the results evidenced that *trans* isomer **5** is more active than *cis* isomer **3**. In addition, another similar complex known as *trans*-[Cu(acr)_2_(5-MeIm)_2_] **7** was subjected to the same tests. We found that it did not influence the mycelium growth, the result being similar with the positive control. Among *cis*-[Cu(acr)_2_(2-EtIm)_2_] **4** and *trans*-[Cu(acr)_2_(2-EtIm)_2_] **6**, it was found that *cis* isomer **4** exhibits fungicidal effect against *Penicillium* sp., this being evidenced by the complete inhibition of mycelium growth [32].

Complex [Co(acr)_2_(HBzIm)_2_]∙0.5H_2_O **8** [33] exhibits activity against *C. albicans*, with an MIC value of 62.5 μg∙mL^−1^. Furthermore, species [Cu_2_(acr)_4_(HBzIm)_2_] **9**, [Cu(acr)_2_(HBzIm)_2_] **60,** and [Cu(acr)_2_(HBzIm)_2_(H_2_O)]∙(H_2_O) **10** have also proven their antifungal properties against *C. albicans,* with MIC ranging between 2 and 62.5 μg∙mL^−1^ [34].

Antifungal activity against *C. albicans* was identified also for [Ni(acr)_2_(HBzIm)_2_(H_2_O)]∙3H_2_O **11,** [Ni(acr)_2_(2-MeBzIm)_2_(H_2_O)]∙1.5H_2_O **12**, [Ni(acr)_2_(5-MeBzIm)_2_(H_2_O)] **15,** and [Ni(acr)_2_(5,6-Me_2_BzIm)_2_] **20,** but the effect was rather modest [35].

Good antifungal activity, sustained by an MIC of 62.5 μg∙mL^−1^, was reported for [Co(acr)_2_(2-MeBzIm)_2_]∙0.5H_2_O **13** and [Co(acr)_2_(5-MeBzIm)_2_] **14** [33].

Moderate activity against *C. albicans* was proven for [Cd(acr)_2_(2,2′-bipy)]∙1.5H_2_O **21** and [Cd(acr)_2_(phen) (H_2_O)] **27**, with an MIC value of 256 μg∙mL^−1^ [37], and there was a good one for [Cu(acr)_2_(2,2′-bipy)(H_2_O)] **22**, [Ni(acr)_2_(2,2′-bipy)(H_2_O)] **23**, [Mn_3_(acr)_6_(2,2′-bipy)_2_] **25,** and [Zn(acr)_2_(2,2′-bipy)]∙H_2_O **26**, with an MIC value of 128 μg∙mL^−1^ [38].

Complexes [Co(macr)_2_(HIm)_2_] **37**, [Co(macr)_2_(2-MeIm)_2_] **38** and [Co(macr)_2_(2-EtIm)_2_] **39** also inhibited the growth of *C. albicans*, with assessed MIC values ranging between 7.8 and 15.6 μg∙mL^−1^. In addition, these complexes were tested as antibacterial agents too (see Section 2.1) and by comparing those results with the antifungal activity presented here it could be considered that the most susceptible microorganism was fungal strain *C. albicans* [43].

Organometallic species [Me_2_Sn(cpma)_2_] **40**, [Bu_2_Sn(cpma)_2_] **41,** [Oct_2_Sn(cpma)_2_] **42**, [Me_3_Sn(cpma)] **43**, [Bu_3_Sn(cpma)] **44**, and [Ph_3_Sn(cpma)] **45** (Hcpma = 3-(4-cyanophenyl)-2-methylacrylate) were screened for antifungal activity against *A. flavus, A. niger, A. fumigatus*, and *Fusarium solani* and, based on the percentage of growth inhibition (PGI) values around compound **41**, presented significant activity against *F. solani* (PGI 95%) [44].

The antifungal activities of *trans*-[Ni(cin)_2_(py)_2_(H_2_O)_2_] **48**, *trans,cis-*[Ni(mcin)_2_(2,2′-bipy)(H_2_O)_2_] **49**, {*trans*-[Ni(mcin)_2_(en)_2_]∙[Ni(en)_2_](mcin)_2_∙H_2_O} **50**, and *trans*-[Ni(ncin)_2_(py)_2_(H_2_O)_2_] **51** were investigated recently. Single-crystal X-ray diffraction revealed species a mononuclear structure for all, unidentate behavior of carboxylate, and a *trans* configuration of different ligands (Figure 3f–h). Significant antifungal activity (PGI higher than 70%) was noticed in the case of complex **48** against *Mucor piriformis* and of complexes **49** and **51** against *A. niger*. Good antifungal activity (PGI 60–70%) was identified for complex **49** against *M. piriformis* and *Helminthosporium solani* and for complex **51** against *H. solani* [49].

A complex containing p-coumaric acid (p-hydroxicinnamic acid), formulated [Zn_4_(HCou)_8_(H_2_O)_6_]∙4H_2_O **52**, was found to present good activity against *C. albicans,* with a PGI value of 63% [53].

In addition, [Zn(Hfer)_2_]*·*1.5H_2_O **53** (H_2_fer = ferulic acid or 3-methoxy-4-hydroxicinnamic acid) [54] inhibits the growth of *C. albicans*. This behavior is sustained by a PGI of 98.9%, which is higher than the level of ferulic acid or sodium ferulate.

Going further, organometallic species [Me_2_Sn(hmpp)_2_] **54**, [Bu_3_Sn(hmpp)] **61** (Bu = n-butyl), [Ph_3_Sn(hmpp)] **62** (Ph = phenyl) presented good antifungal activity against *A. flavus* with a PGI of 60–70%, and against *Microsporum canis* with a PGI of around 60% [58].

The evaluation of the antifungal activity of [Co(mal)(2-apy)] **56** and K[Co(mal)(8-hqn)] **59** evidenced the good activity of **56** against *Bipolaris sorokiniana* (IZD 26 mm) in comparison with **56** (IZD 22 mm). In addition, complex **59** presented very good antifungal activity against *Trichophyton* with an IZD of 44 mm [64].

The analysis of the scarce literature data related to fumarate complexes with biological properties revealed complex [Mn_2_(fum)_2_(phen)_2.5_]∙3H_2_O **63** (H_2_fum = fumaric acid), which presents good antifungal activity against *C. albicans* with a PGI of 69%. Furthermore, it has been found that the antifungal potential is related with 1,10-phenantroline rather than fumarate one [65].

## 3. Coordinative Compounds with Unsaturated Carboxylate Developed for Antitumor Applications

Cancer is one of the most insidious diseases of this century. This comes from its manifestation in various forms and its ability to develop metastases and to adapt to available drugs by developing acquired resistance. These aspects require not only the finding of new drugs, combinatory therapies, and efficient antitumor drug carriers, but also the development of efficient species for both metastases and resistant tumor treatment.

Among the current species under clinical testing, the complexes seem to be a valuable choice in view of their multiple mechanisms of action and good activity against a great number of tumors [66].

Unmistakable, the available platinum anticancer drugs (cisplatin, carboplatin, and oxaliplatin) still play an important role in cancer treatment [67]. However, they exhibit severe side effects and as a result both prescribed doses and effectiveness are limited to a small number of tumor cells. Most importantly, all develop intrinsic resistance [68,69,70]. Interestingly, cisplatin, and oxaliplatin also exhibit cancer immunomodulatory functions and the ability to induce immunogenic cell death (ICD) [66,71].

Recent findings indicate that cisplatin and its analogs effectively inhibit the differentiated tumor cells, but not the cancer stem cells (CSCs) ones. It was shown that if a very small number of such cells remained after treatment, these could lead to resistance, metastases, and relapse; these aspects all diminish the drug’s effectiveness [72].

A selection of the most active antitumor species is presented in Table 2.

### 3.1. Coordinative Compounds with Unsaturated Carboxylate with Antitumor Activity

The experience gained in Pt(II)-based antitumor agents indicated that the use of the advantageous properties of octahedral Pt(IV) complexes was an efficient approach in developing effective agents for treating resistant tumor cells. On the other hand, the design of such compounds also enables their conjugation in axial positions with diverse biologically active ligands to achieve both improved and targeted delivery [70,72,73,74,75,76]. Thus, a combinatory therapy can be developed through both the ligands and Pt(II)-species after accumulation and the reduction of the complex inside the tumor cells, where they act via several mechanisms on different cellular targets [77,78]. As a result, such conjugates can carry both the platinum component and other antitumor components into the tumor cells, some being recognized specifically by certain cells.

To develop species able to inhibit both the proliferation of the differentiated tumor cells and CSCs, some Pt(IV) prodrugs with axial cinnamate ligands were synthesized. This series of complexes was designed by considering the ability of cinnamic acid to reduce the tumorigenic ability of CSCs [73]. Complexes cis,trans,cis-[Pt(NH_3_)_2_(OH)(cin)Cl_2_] **64** and cis,trans,cis-[Pt(NH_3_)_2_(cin)_2_Cl_2_] **65** exhibit enhanced antiproliferative activity in both monolayer and 3D spheroid assays in cervical adenocarcinoma (HeLa), colon carcinoma (HCT116, both p53-positive and p53-non-expressing), breast cancer (invasive ductal carcinoma) (MDA-MB-231), adenocarcinoma (MCF-7), and muscle rhabdomyosarcoma. It is worth mentioning the enhanced activity in comparison with the cisplatin of both species, with **65** also being more active in comparison to **64**. The released Pt(II) compound inhibits cancer cells via the DNA damage mechanism and, moreover, the cinnamic acid, thus liberated, makes the CSCs more sensitive to platinum [73].

On the other hand, the species *cis*,*trans*,*cis*-[Pt(NH_3_)_2_(cin)(ole)Cl_2_] **66** was proved to be active on a series of tumor cell lines, with the overexpression of epidermal growth factor receptor 2 (HER2) via breast cancer lines MCF-7, T47D, MDA 453, and SK-BR-3. Data indicate that the sensitivity of cells toward **66** correlated with the level of HER2 expression, with the highly HER2-expressing SK-BR-3 cells being significantly (4.4-fold) more sensitive than HER2-non-expressing MCF-7 cells [79].

Prodrug *cis*,*trans*,*cis*-[Pt(NH_3_)_2_(cin)(val)Cl_2_] **67** (Hval = valproic acid) was constructed to develop antiproliferative triple action against human lung (A549), breast (MCF-7), hepatocellular (HepG-2), bladder (5637), mice bladder (MB49), and breast (4T1) carcinoma. This complex, bearing a cisplatin moiety and cin and val as axial ligands, acts synergistically via DNA damage, MMP-2 and -9 activity inhibition, tumor cell invasion, and metastasis blocking, suppressing HDAC activity to increase the accessibility of DNA to Pt(II) moiety by decondensing chromatin. Its activity against all cell lines was enhanced in comparison with cisplatin, its antiproliferative activity being evidenced at submicromolar concentrations [80].

The Pt(IV) prodrug *cis*,*trans*,*cis*-[Pt(NH_3_)_2_(fer)_2_Cl_2_] **68,** developed by starting from ferulic acid, was screened against lung carcinoma (A549 and A549/DDP) cells and was found to be more active than cisplatin, acting at a submicromolar level [81]. Moreover, organometallic species [Bu_3_Sn(fer)] **69** was able to reduce the viability of human colon cancer cells (HCT116, HT-29 and Caco-2) at a nanomolar range. The cell viability reduction induced by **69** was associated with G2/M cell cycle arrest, increases in membrane permeabilization, and the appearance of typical morphological signs, like autophagic proteins, that finally trigger the cell death process. As a result, this derivative represents a promising therapeutic agent for colon cancer since it is able to trigger autophagic cell death, an important aspect for overcoming resistance [82].

Compared with other species with cinnamate, the complex [Mg(cin)_2_(H_2_O)_2_]_n_ **70** adopts a layered structure with a pseudooctahedral coordination around the metal center and exhibits very low cytotoxicity against neoplastic A549 (lung), MCF-7 (breast), P388 (murine leukemia), and normal BALB3T3 (mouse fibroblasts) cell lines. In silico parameter calculations indicate good lipophilicity, which suggests this species has an optimal pharmacokinetic profile [83]. Some lanthanide compounds, namely, [M(cin)_3_] **71** (M = Y, La, Ce, Nd, Sm, Yb), [La(4-OMecin)_3_]·2H_2_O **72**, and [La(4-Clcin)_3_]·2H_2_O **73** (4-HOMecinn = 4-methoxicinnamic acid; Clcin = 4-chlorocinnamic acid), contain cinnamate-derived anions, which act as bidentate chelate ligands in all complexes. Unfortunately, activity against HL60 (human promyelocytic leukemia), K562 (human erythromyeloblastoid leukemia), and MCF7 (breast cancer) cell lines was rather very modest [84].

In the series of monomeric organometallic species bearing cinnamate moieties [Sb(p-tolyl)_3_(fnacr)_2_] (Hfnacr = 3-(furan-2-yl)acrylic acid) **74**, [Sb(p-tolyl)_3_(mfnacr)_2_] (Hmfnacr = 3-(5-methylfuran-2-yl)acrylic acid) **75**, and [Sb(p-tolyl)_3_(tfacr)_2_] **76** (Htfacr = 3-(thiophen-2-yl)acrylic acid), antimony (V) was found to adopt a distorted trigonal–bipyramidal geometry, with p-tolyl groups in equatorial positions and carboxylate in axial positions. These species were found to display modest cytotoxicity against hepatocellular carcinoma (HepG2) cells, with IC_50_ values of 7.44, 4.61, and 5.0 mg∙mL^−1^, respectively [85].

**Figure 4:**
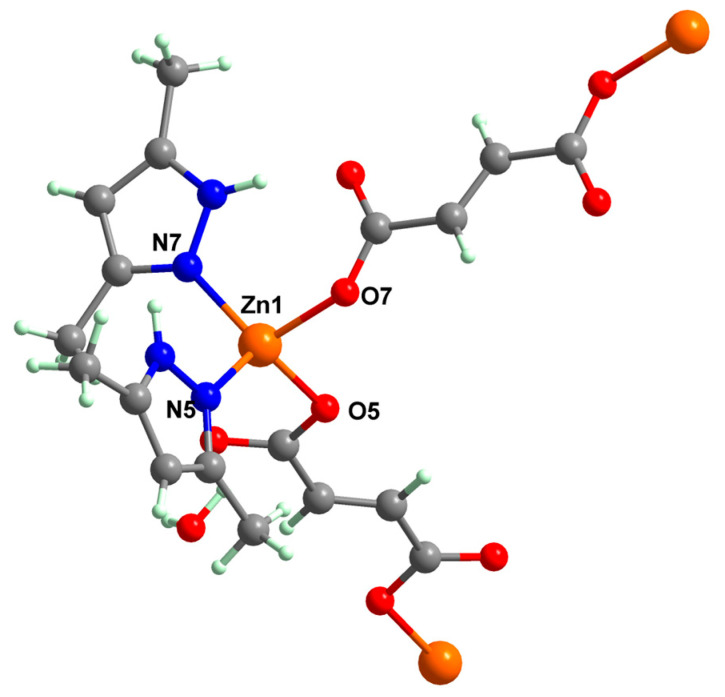
Molecular structures of {[Zn_2_(μ-fum)_2_(Hdmpz)_4_]∙3H_2_O}_n_ (**77**) [86].

Fumarate bridged Zn(II) coordination polymer {[Zn_2_(μ-fum)_2_(dmpz)_4_]·3H_2_O}_n_ **77** was synthesized and structurally characterized by single-crystal X-ray diffraction (Figure 4). The zig-zag polymeric chain of **77** parts self-assembles into a 2D supramolecular network via noncovalent CH⋯π, CH⋯C, NH⋯O and CH⋯O interactions. The compound exhibits cytotoxicity in Dalton’s lymphoma (DL) malignant cancer cell line in a micromolar range, with activity enhanced compared to cisplatin [86].

**Table 2:** Coordination compounds of unsaturated carboxylates with antitumor activity.

Compound Structure */Formulation	Unsaturated Carboxylate Ligand	Auxiliary Ligand	Biological Activity	Ref.
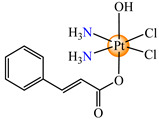 *cis*,*trans*,*cis*-[Pt(NH_3_)_2_(OH)(cin)Cl_2_] (**64**)	cinnamate	ammonia, hydroxyl, chloride	HeLa, HCT116 (p53 positive and p53 non-expressing), MDA-MB-231, MCF-7, rhabdomyosarcoma human cells (IC_50_ in micromolar range)	[73]
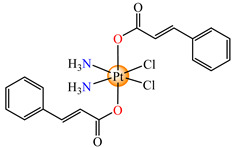 *cis*,*trans*,*cis*-[Pt(NH_3_)_2_(cin)_2_Cl_2_] (**65**)	cinnamate	ammonia, chloride	HeLa, HCT116 (p53 positive and p53 non-expressing), MDA-MB-231, MCF-7, rhabdomyosarcoma human cells (IC_50_ in submicromolar range)	[73]
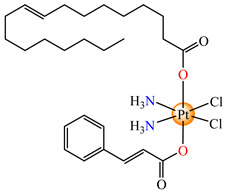 *cis*,*trans*,*cis*-[Pt(NH_3_)_2_(cin)(ole)Cl_2_] (**66**)	cinnamate, oleate	ammonia, chloride	MCF-7, T47D, MDA 453 and SK-BR-3 breast cancer cells (IC_50_ in micromolar range)	[79]
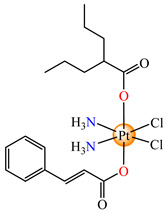 *cis*,*trans*,*cis*-[Pt(NH_3_)_2_(cin)(val)Cl_2_] (**67**)	cinnamate	ammonia, valproate, chloride	human lung (A549), breast (MCF-7), hepatocellular (HepG-2), bladder (5637), mice bladder (MB49) and breast (4T1) carcinoma (IC_50_ in submicromolar range)	[80]
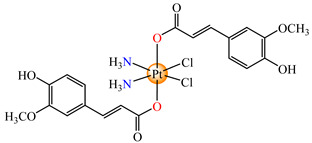 *cis*,*trans*,*cis*-[Pt(NH_3_)_2_(fer)_2_Cl_2_] (**68**)	ferulate	ammonia, chloride	lung carcinoma (A549 and A549/DDP) (IC_50_ in submicromolar range)	[81]
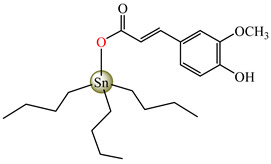 [Bu_3_Sn(fer)] (**69**)	ferulate	butyl	HCT116, HT-29 and Caco-2 (IC_50_ in nanomolar range)	[82]
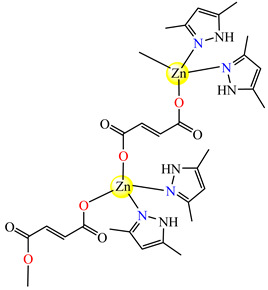 {[Zn_2_(μ-fum)_2_(Hdmpz)_4_]∙3H_2_O}_n_ (**77**)	fumarate (*bridge*) *	3,5-dimethylpyrazole	DL (IC_50_ in micromolar range)	[86]

* carboxylate coordination mode evidenced by X-ray structure.

Of note, the introduction of unsaturated carboxylate ligand(s) into the axial position of the Pt(IV) derivatives of cisplatin or in organotin species componence led to significant activity for some selected tumor cell lines with known poor sensitivity to platinum drugs. These results support the dual action of such compounds through a synergistic mechanism, involving both the effects of cisplatin/organotin species and of unsaturated carboxylate moieties, thus leading together to the tumor cells apoptosis.

### 3.2. Coordinative Compounds with Unsaturated Carboxylate with Antitumor Activity on Resistant Cells

For efficient drug delivery and high therapeutic effects towards resistant cancer tumors, multimodal chemotherapy and immunotherapy were developed. This led to an interesting nanoformulation, generated based upon the self-assembly of a polymerizable *cis*,*trans*,*cis*-[Pt(NH_3_)_2_(icemac)_2_Cl_2_] **78** (Hicemac = 2-isocyanatoethyl methacrylic acid) complex with a thermosensitive polymer with azo bonds that can generate radicals upon exposure to heat. The nanoparticles exhibit a higher cellular uptake than the molecular drug cisplatin and, upon exposure to irradiation, the azo bonds are broken, and therapeutically active species are released. Due to the presence of reactive acrylate moieties, the complex undergoes in situ polymerization inside the cancer cells, resulting in the formation of a cross-linked polymeric network. After that, the reduction to cisplatin into cancer cells triggers cell death via a combination of apoptosis and immunogenic cell death. The therapeutic efficiency of the system was evaluated in a clinical ovarian cancer patient-derived xenograft mouse model as well as in SKOV3DDP, SKOV3, and ES2 ID8 subcutaneous tumor-bearing C57BL/6 mouse models [87].

## 4. Coordinative Compounds with Unsaturated Carboxylate with DNA-Intercalative Abilities and Antioxidant Activity

The interactions of coordinative compounds with DNA have been an active area of research in the last years since these biomolecules represent the primary intracellular targets of antitumor drugs. As a result, complexes with DNA-intercalative properties can be further studied in order to develop new species for cancer treatment [88,89,90,91,92,93,94,95].

In this regard, some complexes with unsaturated carboxylates and an ancillary ligand with aromatic rings or heterorings, able to establish π-π stacking interactions with purine or pyrimidine bases of nucleic acids, were designed.

A selection of the most active species is depicted in Table 3.

**Figure 5:**
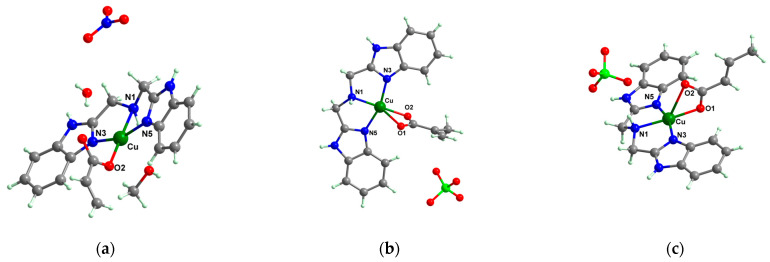

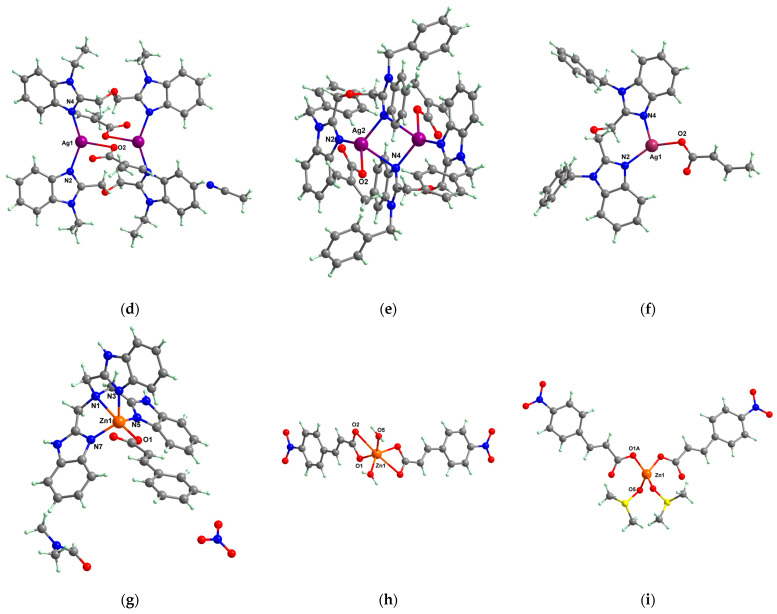
Molecular structures of [Cu(acr)(bba)(CH_3_O)]NO_3_∙H_2_O **79** (**a**) [96], [Cu(macr)(bba)]ClO_4_ **80** (**b**) [96], [[Cu(crot)(bba)]ClO_4_ **81** (**c**) [96], [Ag_2_(macr)_2_(etobb)_2_]∙CH_3_CN **82** (**d**) [98], [Ag(macr)(bobb)] **83** (**e**) [98], [Ag(crot)(bobb)] **85** [99] (**f**), [Zn(cin)(tbima)]NO_3_∙DMF **88** [102] (**g**), [Zn(ncin)_2_(H_2_O)_2_] **89** [103] (**h**), and [Zn(ncin)_2_(DMSO)_2_] **90** [103] (**i**).

Such abilities were evidenced for ternary complexes [Cu(acr)(bba)](NO_3_)⋅H_2_O **79**, [Cu(macr)(bba)]ClO_4_ **80**, and [Cu(crot)(bba)]ClO_4_ **81** (bba = bis(2-benzimidazolylmethyl)amine). The structure consists of cationic species with Cu(II) in a square–pyramidal geometry, constructed using amine as tetradentate and carboxylate as chelate, excepting **79,** where both carboxylate and methanol act as unidentate ligands (Figure 5a–c). The interaction of these species with calf thymus DNA was investigated via electronic absorption, fluorescence spectroscopy and viscosity measurements, and all suggest that both the ligand and complexes bind to DNA in an intercalation mode. The DNA-binding affinity follows the order **80** > **81** > **79** > bba, and this behavior can arise from the large coplanar aromatic rings in the benzimidazole structure [96].

Antioxidants or free radical scavengers are species that can reduce or prevent the cellular damage generally caused by reactive oxygen species (ROS). Since there are various neurological disorders based on the mechanism of oxidative injury associated with free radicals, their scavengers are useful for the prevention and delaying of neurological disorders [97]. As antioxidant activity, the superoxide radical scavenging ability of the above complexes has been investigated indirect through nitroblue tetrazolium (NBT) method. The results indicate that all complexes exhibit superoxide radical scavenging activity in a micromolar range [96].

The same ability was evidenced for [Ag_2_(macr)_2_(etobb)_2_]·CH_3_CN **82**, [Ag(macr)(bobb)] **83**, and [Ag_2_(macr)_2_(aobb)]_n_ **84** (etobb = 1,3-bis(1-ethylbenzimidazol-2-yl)-2-oxapropane, bobb = 1,3-bis(1-benzylbenzimidazol-2-yl)-2-oxapropane, aobb = 1,3-bis(1-allylbenzimidazol-2-yl)-2-oxapropane). In binuclear complexes **82** and **83**, each Ag(I) atom is surrounded by one unidentate methacrylate, while the benzimidazole derivative acts as bridge (Figure 5d,e). Compound **82** displays the highest intercalative ability and **83** has the best ability to scavenge hydroxyl radical [98].

By using sodium crotonate, species [Ag(crot)(bobb)] **85** [99], [Ag(crot)(bebt)] **86** (bebt = 1,3-bis(1-ethylbenzimidazol-2-yl)-2-thiapropane) [100] and [Ag_2_(crot)_2_(aobb)]_n_ **87** [101] were designed for the same purpose. The structural characterization of **85** revealed a mononuclear three-coordinate configuration, in which carboxylate acts as a unidentate and benzimidazole derivative as a chelate (Figure 5f) [99].

Compound [Zn(tbima)(cinn)]NO_3_⋅DMF **88** (tbima = tris(2-benzimidazylmethyl)amine) was also synthesized and characterized as both the DNA intercalator and hydroxyl radical scavenger. Single-crystal X-ray diffraction revealed that Zn(II) is five-coordinated in a distorted trigonal–bipyramidal geometry, with cinnamate serving as a unidentate and amine as a tetradentate chelate (Figure 5g) [102].

The strong electrostatic DNA binding properties which may be employed in the design of new drugs were revealed also for complexes [Zn(ncin)_2_(H_2_O)_2_] **89** and [Zn(ncin)_2_(DMSO)_2_] **90.** Metallic ions adopt a distorted octahedral stereochemistry for **89** and tetrahedral one for **90**, while carboxylate ligands act as a bidentate in both species, as X-ray diffraction analysis revealed (Figure 5h,i) [103].

**Table 3:** Coordination compounds with unsaturated carboxylates with DNA binding ability and antoxidant activity.

Compound Structure */Formulation	Unsaturated Carboxylate Ligand	Auxiliary Ligand	Biological Activity	Ref.
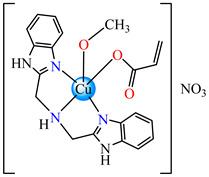 [Cu(acr)(bba)(CH_3_O)]NO_3_∙H_2_O **79**	acrylate (*unidentate*) *	bis(2-benzimidazolylmethyl)amine, methanol	DNA binding, superoxide radical scavenger (IC_50_ = 1.55 mM)	[96]
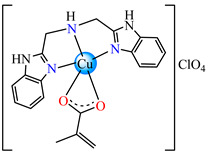 [Cu(macr)(bba)]ClO_4_**80**	methacrylate (*chelate*) *	bis(2-benzimidazolylmethyl)amine	DNA binding, superoxide radical scavenger (IC_50_ = 0.87 mM)	[96]
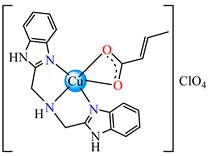 [Cu(crot)(bba)]ClO_4_**81**	crotonate (*chelate*) *	bis(2-benzimidazolylmethyl)amine	DNA binding; superoxide radical scavenger (IC_50_ = 1.27 mM	[96]
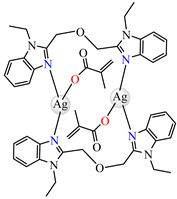 [Ag_2_(macr)_2_(etobb)_2_]∙CH_3_CN **82**	methacrylate (*unidentate*) *	1,3-bis(1-ethylbenzimidazol-2-yl)-2- oxapropane	DNA binding	[98]
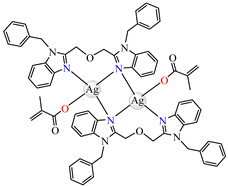 [Ag(macr)(bobb)] **83**	methacrylate (*unidentate*) *	1,3-bis(1-benzylbenzimidazol-2-yl)-2- oxapropane	DNA binding; hydroxyl radical scavenger	[98]
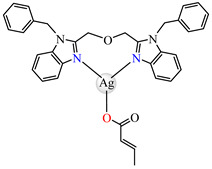 [Ag(crot)(bobb)] **85**	crotonate (*unidentate*) *	1,3-bis(1-benzylbenzimidazol-2-yl)-2- oxapropane	DNA binding; hydroxyl radical scavenger	[99]
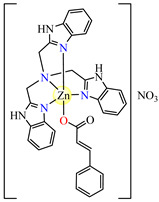 [Zn(cin)(tbima)]NO_3_∙DMF **88**	cinnamate (*unidentate*) *	tris(2-benzimidazylmethyl)amine	DNA binding; hydroxyl radical scavenger	[102]
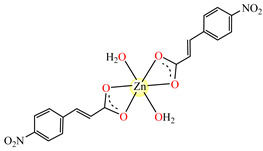 [Zn(ncin)_2_(H_2_O)_2_] **89**	p-nitro cinnamate (*chelate*) *	water	DNA binding	[103]
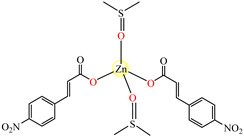 [Zn(ncin)_2_(DMSO)_2_] **90**	p-nitro cinnamate(*unidentate*) *	dimethylsulfoxide	DNA binding	[103]

* carboxylate coordination mode evidenced by X-ray structure.

## 5. Conclusions

Some unsaturated carboxylates, such as acrylate, methacrylate, fumarate, maleate, cinnamate, ferulate, coumarate, and itaconate, often accompanied by N-based heterocyclic species (pyridine, imidazole, and pyrazole derivatives), were used to generate valuable compounds with biological properties. Thus some complexes, mostly made with metal ions from 3D series (Cu(II), Co(II), Ni(II), Mn(II) and Zn(II)), were observed to assess their ability to inhibit resistant strains or develop microbial biofilms on inert surfaces. In addition to enhanced antitumor activity based on a dual-action mechanism, involving both the effects of cisplatin/organotin species and of unsaturated carboxylate moieties, antitumor activity was evidenced against resistant cells. Moreover, the ability to intercalate into DNA strands as well as to scavenge the ROS species was also evidenced for some species, suggesting their future use as antitumor or anti-inflammatory drugs. It is obvious that the trend in this field is to use mainly essential ions for generating biologically active species in order to minimize the toxicity observed for Pt(IV) and Sn(IV). Unmistakably, these activities cannot be directly connected with the presence of unsaturated carboxylates, but this contributes to stereochemistry, stability, water solubility and lipophilicity. All these attributes account for certain biological behaviors. Regarding the synthesis of compounds with mixed ligands (unsaturated carboxylate and N-donor based systems), it is worth mentioning that it was fulfilled for the majority of species, going through a general two-step protocol. First, this included the preparation of carboxylate derivatives, either from metal oxide/carbonate in reaction with carboxylic acid or from a salt in reaction with sodium carboxylate, followed by the addition of N-donor ligand in a proper ratio. Instead, for Sn(IV) derivatives, the synthetic protocol consists in mixing the corresponding organotin chloride, either with silver or sodium salt of carboxylate.

## 6. Further Perspectives

The interest in complexes bearing unsaturated carboxylates could arise from their ability to generate metal-containing polymers with medical applications. Although several metal containing-monomers with unsaturated carboxylates exhibit valuable antimicrobial and antitumor properties, none were studied in order to generate polymers, so this research direction represents an open field for scientists. There are several reports concerning the polymerization of such compounds, but this process is performed at higher temperatures when complex decomposition occurs, resulting in either nano-oxide or nanometal particles [104,105]. It may be considered that a polymerization method in mild conditions should perhaps be developed for such species in order to preserve the core of the coordination compound. Compounds with good biological activity can also be tested for the inhibition of microbial biofilms or resistant tumors, aspects that have been poorly investigated. In addition, according to our knowledge, the preclinical tests for such compounds have not been reported so far.

## Abbreviations

ABA	antibacterial activity
AFA	antifungal activity
aobb	1,3-bis(1-allylbenzimidazol-2-yl)-2-oxopropane
apy	aminopyridine
ATCC	American Type Culture Collection
bba	bis(2-benzimidazolylmethyl)amine
bebt	1,3-bis(1-ethylbenzimidazol-2-yl)-2-thiapropane
bipy	bipyridine
bobb	1,3-bis(1-benzylbenzimidazol-2-yl)-2-oxapropane
Bu	n-butyl
DMF	*N*,*N*-dimethylformamide
dmpz	3,5-dimethyl-1H-pyrazole
DMSO	dimethylsulfoxide
en	ethylenediamine
Et	ethyl
EtIm	ethylimidazole
Etobb	1,3-bis(1-ethylbenzimidazol-2-yl)-2-oxapropane
H_2_cou	coumaric acid
H_2_fer	ferulic acid
H_2_fum	fumaric acid
H_2_mal	maleic acid
Hacr	acrylic acid
HBzIm	benzimidazole
Hcin	cinnamic acid
HClcin	chlorocinnamic acid
Hcpma	3-(4-cyanophenyl)-2-methylacrylic acid
Hcrot	crotonic acid
Hhmpp	3-(4-hydroxy-3-methoxyphenyl)-2-phenylpropenoic acid
HIm	imidazole
Hicemac	2-isocyanatoethyl methacrylic acid
Hmacr	methacrylic acid
Hmcin	methylcinnamic acid
HMeOcin	methoxy cinnamic acid
Hncin	para-nitro cinnamic acid
Hole	oleic acid
Hpaa	2-phenyl-3-thiophen-2-yl-acrylic acid
Hpz	1H-pyrazole
hqn	hydroxyquinoline
Htaa	3-thiophen-2-yl-acrylic acid
Hval	valproic acid
iqn	isoquinoline
IZD	inhibition zone diameter
MBEC	minimum biofilm eradication concentration
Me	methyl
Me_2_BzIm	dimethylbenzimidazole
MeBzIm	methylbenzimidazole
MeHpz	methyl-1H-pyrazole
MeIm	methylimidazole
MIC	minimum inhibitory concentration
mlm	melamine
PAA	polyacrylates
PGI	percentage growth inhibition
Ph	phenyl
phen	phenantroline
PMAA	polymethacrylic acid
PMMA	poly(methyl)methacrylates
PPF	poly(propylene)fumarate
py	pyridine
qn	quinoline
ROS	reactive oxygen species
tbima	tris(2-benzimidazylmethyl)amine
tea	triethanolamine
tmeda	*N*,*N*,*N’*,*N’*-tetramethylenediamine
